# Authors’ Reply: Promoting Oral Health Literacy Among UAE Public Sector Employees

**DOI:** 10.2196/67634

**Published:** 2024-11-12

**Authors:** Florence Carrouel, Benjamin du Sartz de Vigneulles, Céline Clément, Virginie-Eve Lvovschi, Elise Verot, Valeria Tantardini, Michel Lamure, Denis Bourgeois, Claude Dussart, Romain Lan

**Affiliations:** 1 Health Systemic Process Laboratory (P2S), UR4129, University Claude Bernard Lyon 1, University of Lyon, 7 rue Guillaume Paradin, Lyon, 69008, France, 33 0478785745; 2 Interpsy Laboratory (UR4432), University of Lorraine, Nancy, France; 3 Research on Healthcare Performance Laboratory (INSERM U1290), University Claude Bernard Lyon 1, University of Lyon, Lyon, France; 4 Hospices Civils of Lyon, Lyon, France; 5 PRESAGE Institute, University Jean Monnet, Saint-Etienne, France; 6 Centre Hospitalier Universitaire de Saint-Etienne (CIC 1408 INSERM), Saint-Etienne, France; 7 Geriatric Rehabilitation and Follow-up Care Service, Centre Hospitalier Universitaire of Rouen, Oissel site, Rouen, France; 8 Anthropologie bio-culturelle, droit, éthique et santé Laboratory (ADES, UMR7268), Centre National de la Recherche Scientifique, Etablissement Français du Sang, Aix Marseille University, Marseille, France

**Keywords:** health literacy, oral health literacy, workplace, civil servant, health promotion, prevention, United Arab Emirates

We would like to extend our gratitude to the author for their insightful comments [1] on our published article, “Promoting Health Literacy in the Workplace Among Civil Servants: Cross-Sectional Study” [2]. Their input significantly enhances discussions around health literacy (HL) and oral health literacy (OHL) in diverse populations and work environments, particularly regarding the United Arab Emirates.

OHL and HL are crucial for improving health outcomes. Our study revealed differences in OHL compared to HL across various professional categories, indicating the need for targeted interventions. However, while our study emphasizes the importance of numeracy within the French civil servant population, it is essential not to overlook other aspects of HL, such as comprehension, decision-making, and access to reliable health information, which are equally critical in promoting overall health [2].

On the other hand, while shared concerns regarding the challenges in replicating such a study in the United Arab Emirates are acknowledged, it is important to underline the differences concerning the populations involved, which would likely influence the implementation and outcomes. First, the United Arab Emirates’ workforce includes many expatriates, with differences in educational background and health care access. This diversity could result in greater disparities in OHL and HL [3], making it more difficult to assess and compare HL across occupational groups, unlike the more homogeneous French cohort. Second, the multilingual environment of the United Arab Emirates adds challenges for HL assessments [4]. Unlike our study, which used standardized, validated French questionnaires to ensure consistency, a study of the UAE population would need tools adapted to various languages and cultures. This complicates survey design and health promotion strategies, as interventions must be tailored to the workforce’s diverse linguistic and cultural needs [4]. Third, the health system in the United Arab Emirates is different from France’s, which is characterized by strong public health insurance and workplace health programs. In the United Arab Emirates, access to health care services seems to depend on factors like employment status, nationality, and income. Health promotion initiatives should be adapted to these differences, with tailored interventions to address the needs of different professional categories and address literacy gaps caused by socioeconomic disparities [5].

In conclusion, implementing a similar study to evaluate OHL and HL in the United Arab Emirates is necessary but requires careful consideration of the cultural environment, unique health care landscape, and access to dental and general health services.

## Abbreviations

HL: health literacy
OHL: oral health literacy
